# Feasibility of performing in-scanner exercise testing and assessing myocardial mechanical reserve in normal volunteers and in patients with cardiomyopathy

**DOI:** 10.1186/1532-429X-16-S1-P255

**Published:** 2014-01-16

**Authors:** Joshua Y Cheng, Jie J Cao, Kathy Halloran

**Affiliations:** 1 Research, St. Francis Hospital the Heart Center, Greenvale, New York, USA; 2 Stony Brook University, Stony Brook, New York, USA

## Background

While cardiac MRI provides invaluable insight into cardiac structure, function and increasingly hemodynamics, it remains to be challenging to assess cardiac response to exercise stress at peak workload. The objective of this study was to examine the feasibility of in-scanner exercise with a supine ergometer and to assess the myocardial mechanical reserve from rest to stress.

## Methods

We prospectively recruited 13 normal volunteers and 10 stable patients with known ischemic or nonischemic cardiomyopathy. All participants underwent cardiac MRI in a 1.5 T scanner (Siemens Avanto). Prior to exercise participants were fit tested to a modified supine ergometer (Lode, the Netherlands) attached to the scanner table to ensure that the body habitus was appropriate for exercising inside the bore. Exercise began outside the bore with workload set at 25 Watts which was increased every 3 minutes by 25 Watts or adjusted to subjects' tolerance. Participants were slide into the bore while exercising when they reached near maximal capacity or at least 75% maximally predicted heart rate. Imaging took place while subjects were exercising. Real-time SSFP cine images of the short axis stack covering the whole heart were acquired with free breathing prior to exercise and again at peak exercise. Myocardial circumferential strain was analyzed using Feature Tracking software (CIM V8.1 by Auckland, New Zealand). Endocardial and epicardial contours were drawn in mid ventricle to assess strain of the whole myocardium and of the endocardium.

## Results

All 23 participants successfully completed in-scanner exercise. Heart rate was increased from 62 bpm to 116 bpm for the normal subjects and 61 bpm to 111 bpm for the patients. Systolic blood pressure increased from 115 mmHg to 147 mmHg for normals and 128 mmHg to 171 mmHg for the patients. While both groups demonstrated significant increase in myocardial and endocardial strain (-16 ± 3% to -20 ± 3%, p < 0.001 and -20 ± 4% to -26 ± 4%, p < 0.001 for normals, -14 ± 4% to -16 ± 5%, p < 0.001 and -18 ± 6% to -22 ± 7%, p < 0.001 for patients, respectively) the increase in myocardial and enocardial strain was much greater in normals (30% and 32%, respectively) than in patients (17% and 20%, respectively).

## Conclusions

It is feasible to perform in-scanner exercise testing and to assess myocardial response at peak exercise. Our preliminary findings suggest that there is improvement of myocardial mechanical performance attributing to exercise effect for all subjects. However, normal subjects have more favorable myocardial mechanical reserve than patients with cardiomyopathy.

## Funding

St. Francis Research Foundation.

